# Mechanics-informed machine learning for predicting mechanical properties of heat-treated multi-metal alloys

**DOI:** 10.1038/s41598-026-50390-9

**Published:** 2026-04-26

**Authors:** Fatah Hadji, Atmane Hadji, D. Belfennache, A. Alami, S. Alomairy, Mustafa Jaipallah Abdelmageed Abualreish, M. Fatmi, M. A. Ghebouli, Aseel Smerat, Murat Yaylacı

**Affiliations:** 1 Fundamental Departments of Science and Technology, University of Jijel, 18000 Jijel, Algeria; 2 Laboratory of Mechanics, Materials and Energetic, University of Bejaia, Faculty of Technology, 0006 Bejaia, Algeria; 3 LISI Laboratory, Computer Science Department, University of A. Boussouf Mila, 43000 Mila, Algeria; 4 Research Center in Industrial Technologies CRTI, P.O. Box 64, 16014 Cheraga, Algiers, Algeria; 5 Laboratory of Process Engineering, Materials and Environment, Faculty of Technology, University of Djillali Liabes, 22000 P.O. Box 89, Sidi Bel Abbes, Algeria; 6 Department of Physics, College of Science, Taif University, 21944 Taif, Saudi Arabia; 7 Department of Chemistry, College of Science, Northern Border University, P.O. Box 1321, 91431 Arar, Saudi Arabia; 8 Research Unit on Emerging Materials (RUEM), University Ferhat Abbas of Setif 1, 19000 Setif, Algeria; 9 Department of Chemistry, Faculty of Sciences, University of M’sila, University Pole, Road Bourdj Bou-Arreiridj, 28000 M’sila, Algeria; 10 Hourani Center for Applied Scientific Research, Al-Ahliyya Amman University, Amman, 19328 Jordan; 11 Department of Civil Engineering, Recep Tayyip Erdogan University, 53100 Rize, Türkiye; 12 Turgut Kıran Maritime Faculty, Recep Tayyip Erdogan University, 53900 Rize, Türkiye

**Keywords:** Machine learning, XGBoost, Heat-treated alloys, Mechanical properties, Ultimate tensile strength, Engineering, Materials science

## Abstract

This study investigates the mechanical behavior and damage evolution of heat-treated multi-metal alloys through an integrated framework combining machine learning and mechanics-based analysis. A comprehensive dataset comprising approximately 1500 alloy samples was analyzed to predict ultimate tensile strength (Su) using various regression models. Among the evaluated algorithms, XG Boost demonstrated superior predictive performance, achieving a coefficient of determination of R^2^ = 0.98 and a root mean square error (RMSE) of 44.26 MPa. To ensure model interpretability and physical consistency, SHAP (SHapley Additive ex-Planations) analysis was employed, revealing that shear modulus (G), Young’s modulus (E), elongation at fracture (A5), Brinell hardness (BHN), and yield strength (Sy) are the most influential parameters governing tensile strength. The results indicate that higher elastic and shear moduli significantly enhance Su, in agreement with fundamental mechanical principles. In addition, key mechanical responses including fatigue crack propagation, energy absorption capacity (up to 2420 J), fracture toughness (KIC ranging from 50 to 120 MPa m^1/2^), and surface hardness gradients were analyzed to provide a comprehensive understanding of deformation and failure mechanisms. The proposed approach offers a robust, physically interpretable, and data-driven tool for predicting and optimizing the mechanical performance of heat-treated metallic alloys.

## Introduction

Multi-metallic materials with diverse compositions are widely used in the fabrication of numerous metallic and hybrid components. Owing to the presence of multiple metallic phases, these materials offer tailored properties that make them suitable for various advanced applications, including jet engine turbine blades, biomedical implants, and microelectronic devices. They are also employed in several industrial sectors, notably in heat exchangers and marine engineering, where high performance and reliability are required^1,3^. Among the various tests performed for each type, the most widely evaluated and critical properties are the mechanical characteristics, particularly high strength, hardness, and corrosion resistance^4,5^. In addition, surface treatment techniques in multi-metal processing play a crucial role in enhancing these properties to achieve optimal mechanical performance. However, these treatments are typically multi-stage and time-consuming^6,7^. Conventional methods for improving mechanical properties typically rely on extensive and complex experimental procedures, which are often time-consuming, costly, and inefficient, particularly when dealing with the intricate interrelationships among different mechanical properties^8,9^. Given that the atomic matrix structure, its inherent properties, and the characteristics of the metallic phases are the principal determinants of material performance, it becomes essential to accurately govern the underlying metallurgical and physical phenomena, including solid-solution formation and phase transformations. Such control is achieved through the precise adjustment of microstructural features, heat-treatment parameters, holding time, applied stress rate, phase evolution, and cooling pathways^10,11^. However, predicting the mechanical properties of materials after heat treatment remains a major challenge due to the complex and nonlinear interactions among alloying elements^12^. Peng Wang et al. studied the effect of the Quenching–Partitioning–Tempering (QPT) process on Ce-microalloyed low-alloy steels. They showed that QPT heat treatment improves phase formation and enhances the strength–ductility balance. The addition of cerium refines the microstructure, increases phase stability, and leads to higher hardness, tensile strength, and toughness compared with conventional treatments^13^. Muhammad Sana et al. proposed a three-step modeling approach to optimize dry turning parameters and enhance tribological performance in metal machining. Using an artificial neural network (ANN) combined with NSGA-II evolutionary optimization, the model achieved excellent predictive accuracy (R^2^ ≈ 1). Under optimal conditions (VCS = 100 m/min, FR = 0.2 mm/rev, DOC = 0.5 mm, INType = Xcel), significant improvements were obtained, including a 354% increase in tool life, enhanced surface quality, and improved chip morphology, demonstrating the effectiveness of integrating machine learning with process optimization^14^. Recent studies have demonstrated the effectiveness of machine learning techniques in predicting material behavior and optimizing manufacturing processes. Interpretable ML frameworks combining XGBoost and SHAP analysis have been successfully applied to predict the stretch formability of magnesium alloys using microstructural and mechanical features^15^. In addition, machine learning models such as random forest, decision trees, and gradient boosting have been used to predict forming limit diagrams (FLD) and fracture limits of sheet metals with high accuracy, often integrated with finite element simulations^16^. Furthermore, machine learning approaches have been applied in hot stamping processes to predict thickness distribution and optimize forming parameters based on data generated from finite element modeling^17^. More recently, coupled ML–FE frameworks have been developed to predict formability under varying conditions such as pre-strain and temperature, demonstrating strong predictive capabilities for forming limit diagrams^18^. However, most existing studies mainly focus on specific targets such as formability limits, thickness distribution, or process optimization, without providing a comprehensive analysis of multiple mechanical properties and their underlying physical mechanisms. Moreover, the integration of machine learning with mechanics-based interpretation remains limited. Therefore, this study aims to develop a mechanics-informed machine learning framework that not only ensures high predictive accuracy but also provides physical interpretability and a deeper understanding of deformation and failure behavior in heat-treated multi-metal alloys. The objective of this study is to develop a reliable framework for predicting the mechanical properties of heat-treated metal alloys using machine learning models. It first looks at a dataset to determine the relationships between mechanical variables, and then it links these relationships to underlying structural and physical mechanisms. The study analyzes several models and finds key features that control alloy behavior in order to determine the most reliable and accurate machine learning model. It assesses the predictions’ compliance with accepted mechanical rules to offer an effective and comprehensible tool for material design and performance optimization.

## Dataset description

This research relies on a structured dataset of approximately 1500 records representing different alloy families, designed to support a detailed evaluation of metallurgical behavior, compositional variability, and performance characteristics. The dataset captures essential descriptors including alloy classification (ferrous and non-ferrous alloys such as copper, nickel, titanium, and magnesium), the dominant base element (Fe, Al, Cu, Ni, Ti, Mg), and associated alloying elements (C, Mn, Cr, Ni, Mo, Al, Zn, V). In addition, it documents key material properties namely mechanical strength, electrical and thermal conductivity, corrosion resistance, and strength-to-weight ratio—summarized in Table 1, along with representative industrial applications across construction, automotive, aerospace, electronics, and biomedical sectors, as presented in Table 2. Temporal and documentary attributes, including creation and update dates, further enhance the dataset’s reliability, consistency, and traceability, enabling robust comparative analysis and informed material selection for engineering applications.

**Table 1:** Symbols and definitions of material properties dataset.

Full name	Description/meaning
(Std) Standard	Standard code for the sample
(ID) Identifier	Identification number of the sample
Material	Type of the material
Heat treatment	Type of thermal treatment applied
(Su)Ultimate tensile strength	Maximum stress a material.
(Sy) Yield Strength	Stress at which plastic deformation begins
(A5) Elongation at fracture	Relative elongation of the specimen at fracture
(Bhn) Brinell hardness number	Hardness value measured by the Brinell method
(E) Young’s modulus	Ratio of tensile stress to tensile strain
(G) Shear modulus	Ratio of shear stress to shear strain
(mu) Poisson’s ratio	Ratio of lateral strain to longitudinal strain
(Ro) Density	Mass per unit volume of the material
(pH) Hydrogen potential	Measure of acidity or alkalinity of the medium
(Desc) Description	Qualitative information about the sample or process
(HV) Vickers hardness	Hardness value measured using the Vickers method

**Table 2:** Types, composition, and applications of metallic alloys dataset.

Main element	Alloying elements	Main characteristics
Iron (Fe)	C, Mn, Cr, Ni, Mo	Good mechanical strength, hardness varies with carbon content
Aluminum (Al)	Cu, Mg, Si, Zn	Lightweight, good thermal and electrical conductivity
Copper (Cu)	Zn, Sn, Ni	Excellent electrical conductivity and corrosion resistance
Nickel (Ni)	Cr, Fe, Mo	High resistance to heat and oxidation
Titanium (Ti)	Al, V, Mo	High strength-to-weight ratio, corrosion resistant
Magnesium (Mg)	Al, Zn, Mn	Very light, moderate strength

This study presents a comprehensive analysis of the application of machine learning techniques for predicting the mechanical properties of heat-treated polymetallic alloys, including hardness, tensile strength, and yield strength, based on experimental and processing parameters such as heat treatment temperature and chemical composition. The selected machine learning models were carefully chosen to represent a diverse range of learning strategies, including tree-based methods (e.g., Random Forest and XGBoost), kernel-based approaches (e.g., Support Vector Machine), and neural network-based models (e.g., Artificial Neural Networks and LSTM). This diversity enables a comprehensive comparison of different modeling paradigms and enhances the robustness of the predictive analysis. Furthermore, although more advanced deep learning models such as recurrent neural networks (RNNs) exist, their performance typically requires large-scale datasets and high computational cost. In this study, the dataset size is relatively moderate, making classical machine learning models more suitable in terms of efficiency, stability, and generalization capability. In addition, the selected models are widely used in materials science applications, ensuring reliable and interpretable results. To provide further insight into the machine learning models used in this study, a brief description of their underlying principles is included. Linear regression models estimate the relationship between input variables and output using a linear formulation of the form y = β₀ + Σβ_i_x_i_. Tree-based models such as Random Forest and XGBoost construct ensembles of decision trees to capture complex nonlinear relationships, where XGBoost optimizes predictions through a regularized objective function that balances model accuracy and complexity. Neural network-based models, including artificial neural networks, rely on interconnected layers of neurons in which each unit performs a weighted summation followed by a nonlinear activation function. Regarding hyperparameter optimization, model tuning was performed using a systematic approach to ensure optimal predictive performance. Key parameters such as the number of trees, learning rate, maximum depth, and regularization terms were adjusted through iterative experimentation. A grid search strategy combined with cross-validation was employed to determine the optimal parameter configuration by minimizing error metrics such as RMSE. This approach ensures a balance between model accuracy and generalization while reducing the risk of overfitting. A range of algorithms was evaluated, from traditional approaches like linear regression to advanced methods including random forests, XGBoost, CatBoost, LightGBM, and deep learning-based multilayer neural networks. Each algorithm exhibits distinct characteristics and advantages that affect its ability to capture the complex nonlinear relationships between input parameters and the resulting mechanical properties. This comparative study highlights the value of integrating traditional statistical models with modern machine learning techniques, such as clustering methods or neural networks, to tackle the intrinsic complexity of metallurgical data, thereby providing a robust framework for data-driven materials design. To improve data transparency, a representative subset of the collected dataset is provided in Appendix. The complete dataset was compiled from multiple open-access literature sources and is available from the corresponding author upon reasonable request.

## Results and discussion

**Fig. 1:**
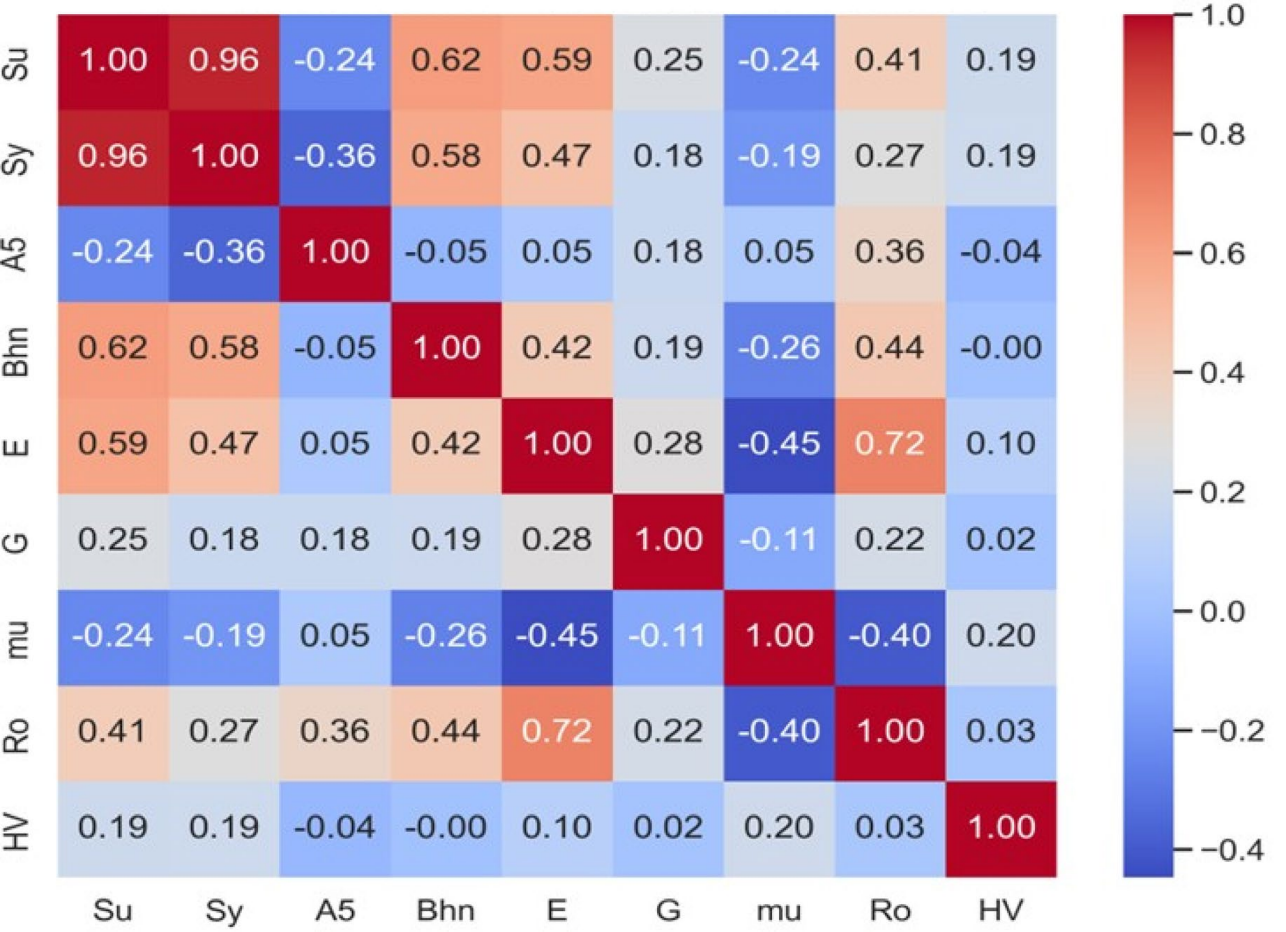
Heatmap of mechanical properties correlation.

**Fig. 2:**
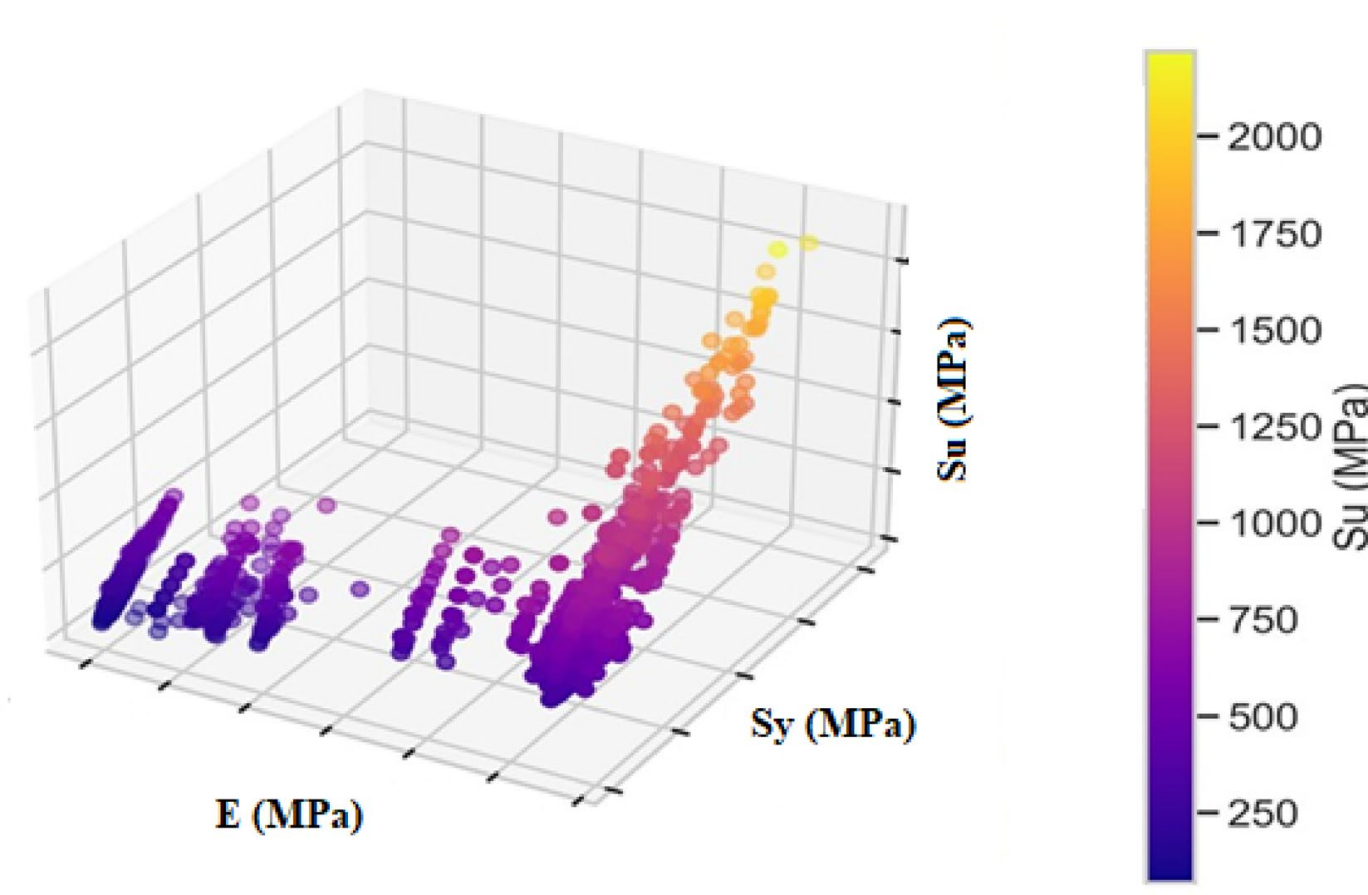
Relationship between elastic modulus, yield strength, and ultimate strength.

Figure 1 presents a heatmap illustrating the correlations among several. Red regions indicate positive correlations, meaning that increases in one property are accompanied by increases in others. This behavior is particularly evident between tensile strength, yield strength, and hardness, which tend to increase simultaneously due to similar strengthening mechanisms induced by heat treatment. The correlation map of the network structures further highlights the relationships between material properties and mechanical response. Notably, strong positive correlations are observed between yield strength and ultimate tensile strength (*r* = 0.92), Young’s modulus and density (*r* = 0.72), Brinell hardness and ultimate tensile strength (*r* = 0.62), as well as between Young’s modulus and ultimate tensile strength. Conversely, blue regions represent negative correlations, where an increase in one parameter corresponds to a decrease in another, reflecting the well-established trade-off between hardness and deformability, commonly observed between strength-related properties and ductility. Strongly correlated parameters convey similar information, and the heatmap facilitates the identification of redundant variables. Moreover, it highlights the key mechanical factors governing the alloy’s behavior after heat treatment, which is essential for selecting the most relevant input variables for machine learning model training. Young’s modulus (E), yield strength (σ_y_), and ultimate tensile strength (σ_u_) are three basic mechanical parameters, and Fig. 2 illustrates their constant distributional connections. According to the observed pattern, materials having a high Young’s modulus typically have greater yield strengths. This suggests a relationship between the initial resistance to plastic deformation and atomic stiffness. The three-dimensional surface also shows that the ultimate tensile strength exhibits a similar pattern, indicating that the alloy’s initial atomic contacts have a significant influence on the behavior up until the point of fracture. This knowledge enables us to distinguish between distinct types of materials (such as brittle materials, hardened alloys, and ductile metals) and assess their structural potential according to their mechanical characteristics.

**Fig. 3:**
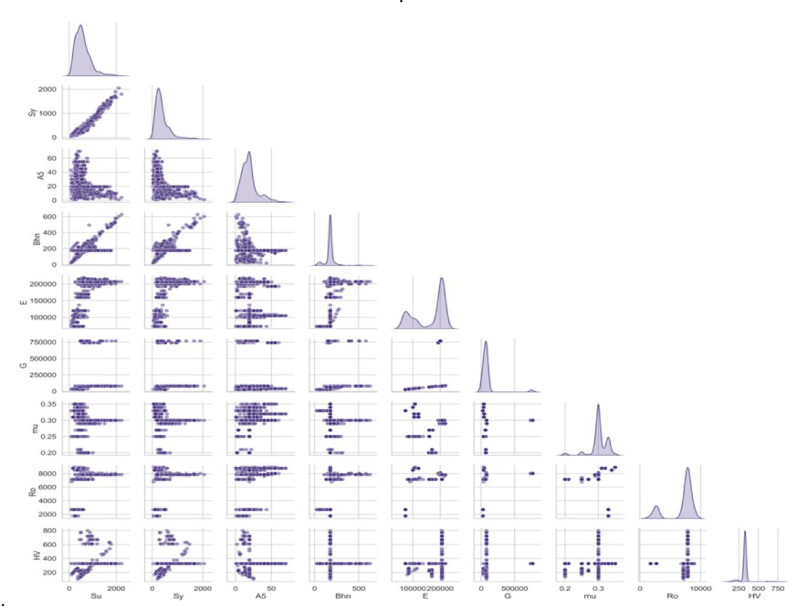
Pair plot of mechanical and microstructural properties.

**Fig. 4:**
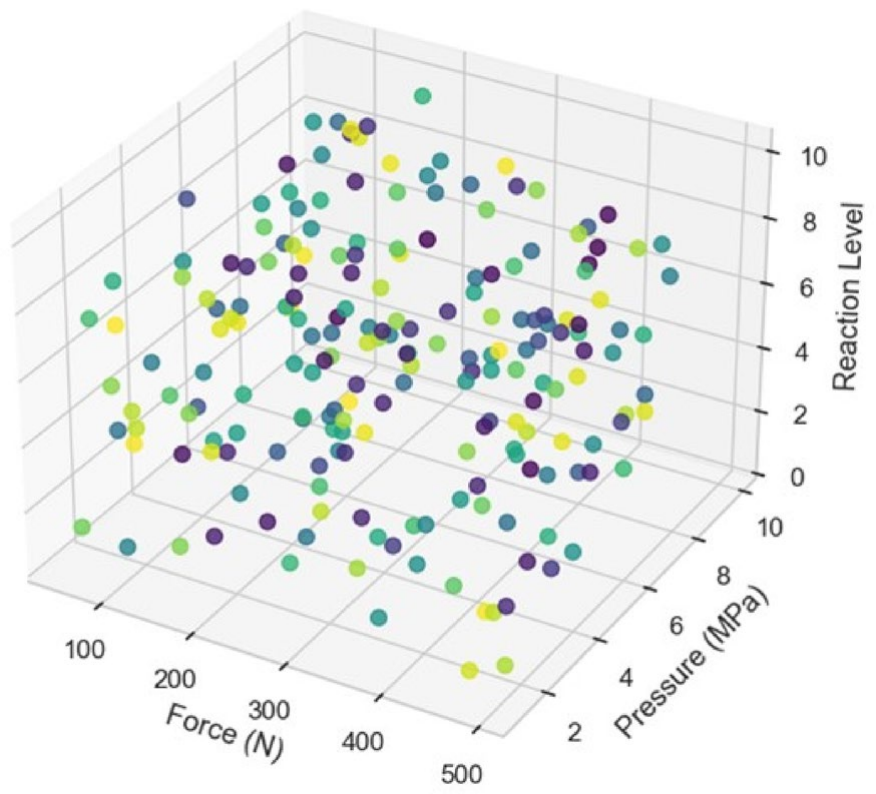
Scatter plot of mechanical property relationships.

Figure 3 presents a pair plot illustrating the distributions and pairwise relationships among mechanical and microstructural properties. The diagonal plots show the individual distributions of each parameter, revealing the dispersion and central tendencies of properties such as ultimate tensile strength (SU), yield strength (SY), elongation at break (A5), hardness (BHN), Young’s modulus (E), shear modulus (G), Poisson’s ratio (µ), density (ρ), and microhardness (HV). The off-diagonal scatter plots highlight the relationships between pairs of variables. Strong positive linear trends are observed between strength-related parameters (SU, SY, BHN, and HV), indicating that increases in strength are accompanied by increases in hardness due to similar strengthening mechanisms. Conversely, elongation (A5) shows weaker or negative correlations with strength and hardness, reflecting the classical trade-off between strength and ductility. The dispersion patterns also suggest the presence of clustering and non-linear relationships for certain properties, emphasizing the complexity of mechanical behavior after processing and the relevance of multivariate analysis. Figure 4 illustrates a three-dimensional distribution of data points revealing a complex, nonlinear interaction between force, pressure, and reaction level. Elevated reaction levels are observed over a broad range of force and pressure values, indicating that the mechanical response is governed by the combined effect of multiple loading parameters rather than by a single dominant variable. Furthermore, the dispersion of the data points reflects the intrinsic variability of the system, which may be attributed to material heterogeneity, microstructural differences, or variations in experimental conditions^19^. This representation facilitates the identification of general trends and regions of enhanced mechanical response and highlights the relevance of data-driven graphical approaches, particularly machine learning techniques, for accurately modeling and predicting complex mechanical behavior^20,21^.

**Fig. 5:**
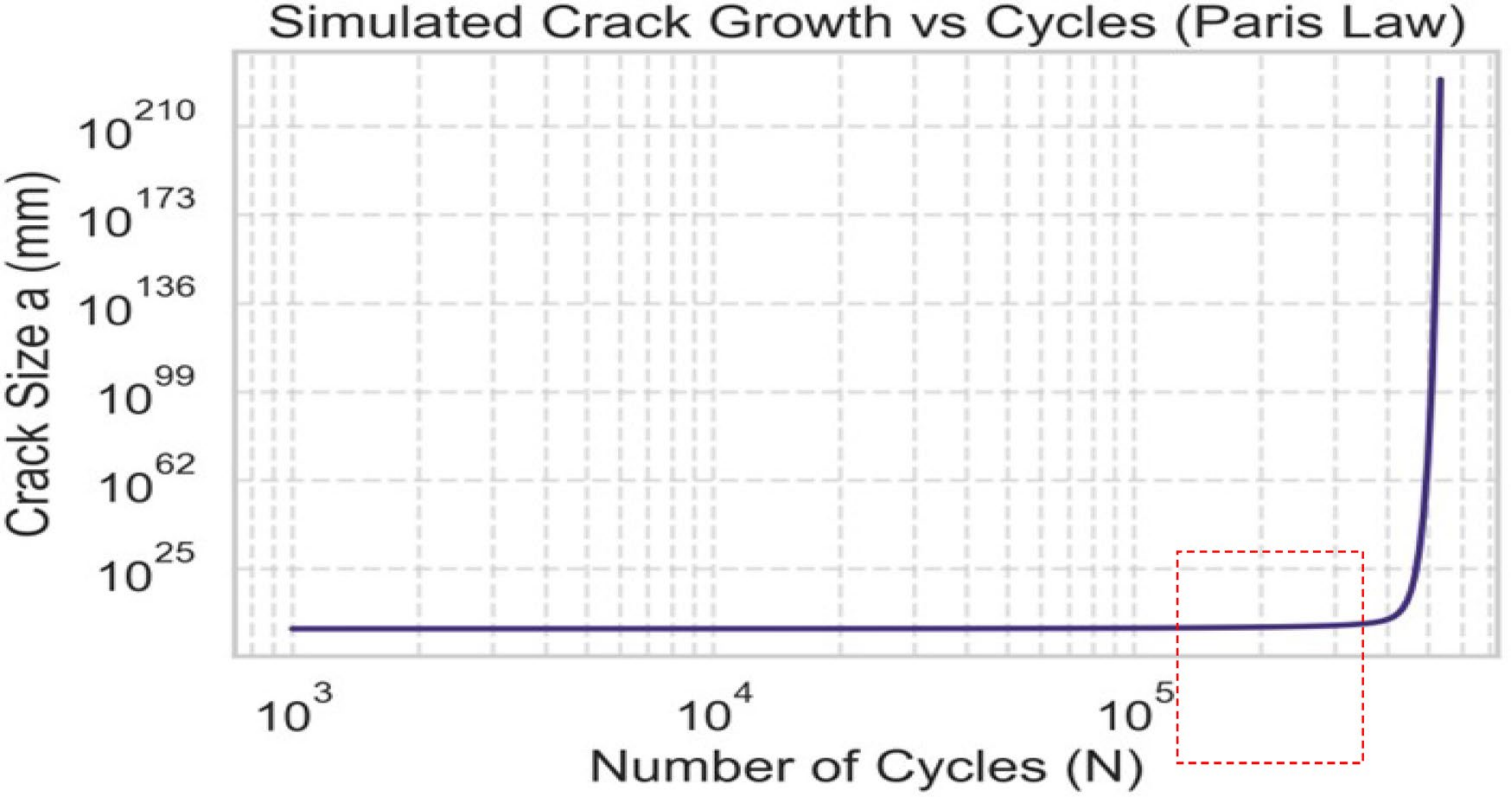
Simulated crack growth as a function of load cycles (Paris’ Law).

**Fig. 6:**
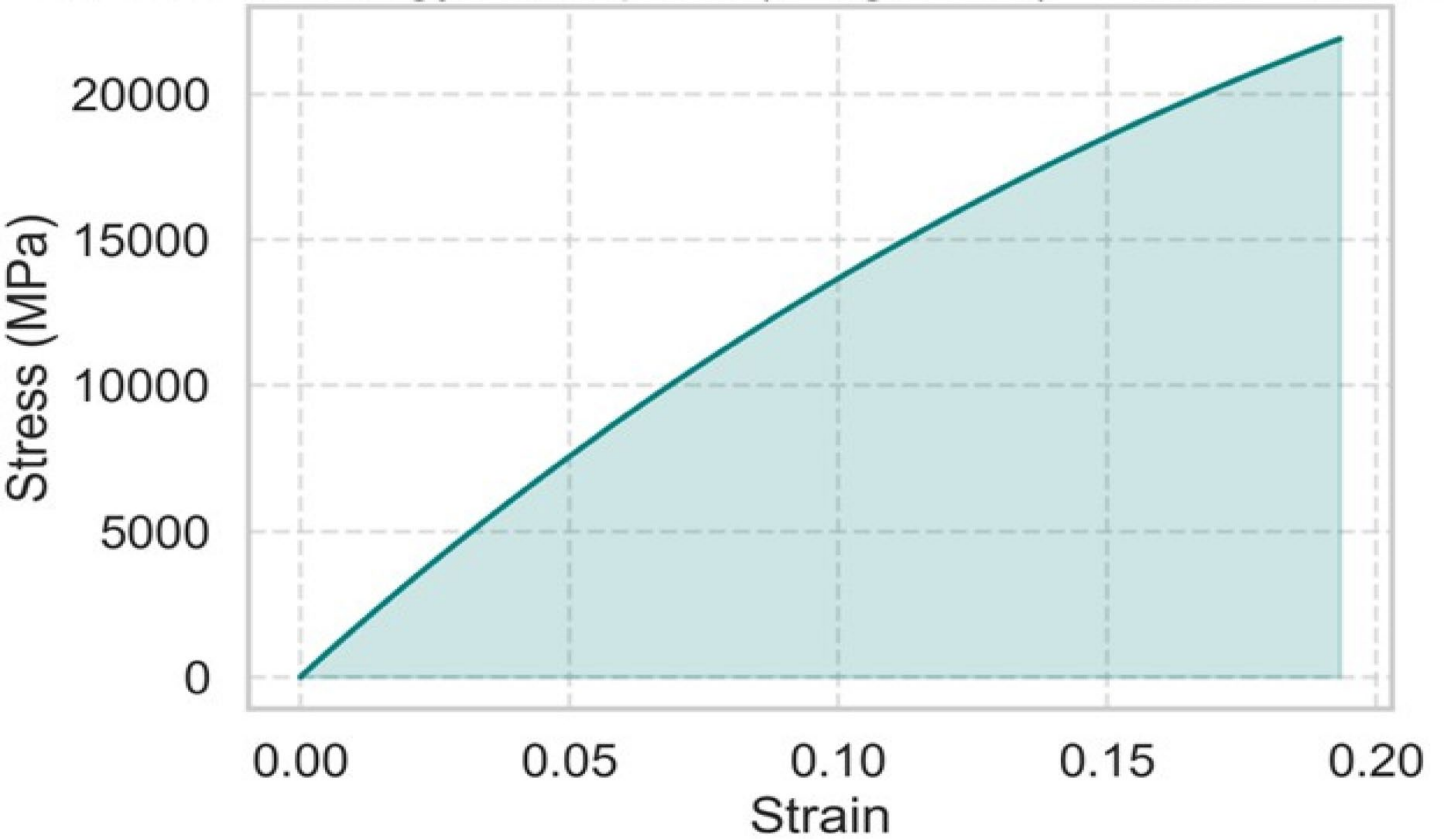
Energy absorption and toughness curve.

**Fig. 7:**
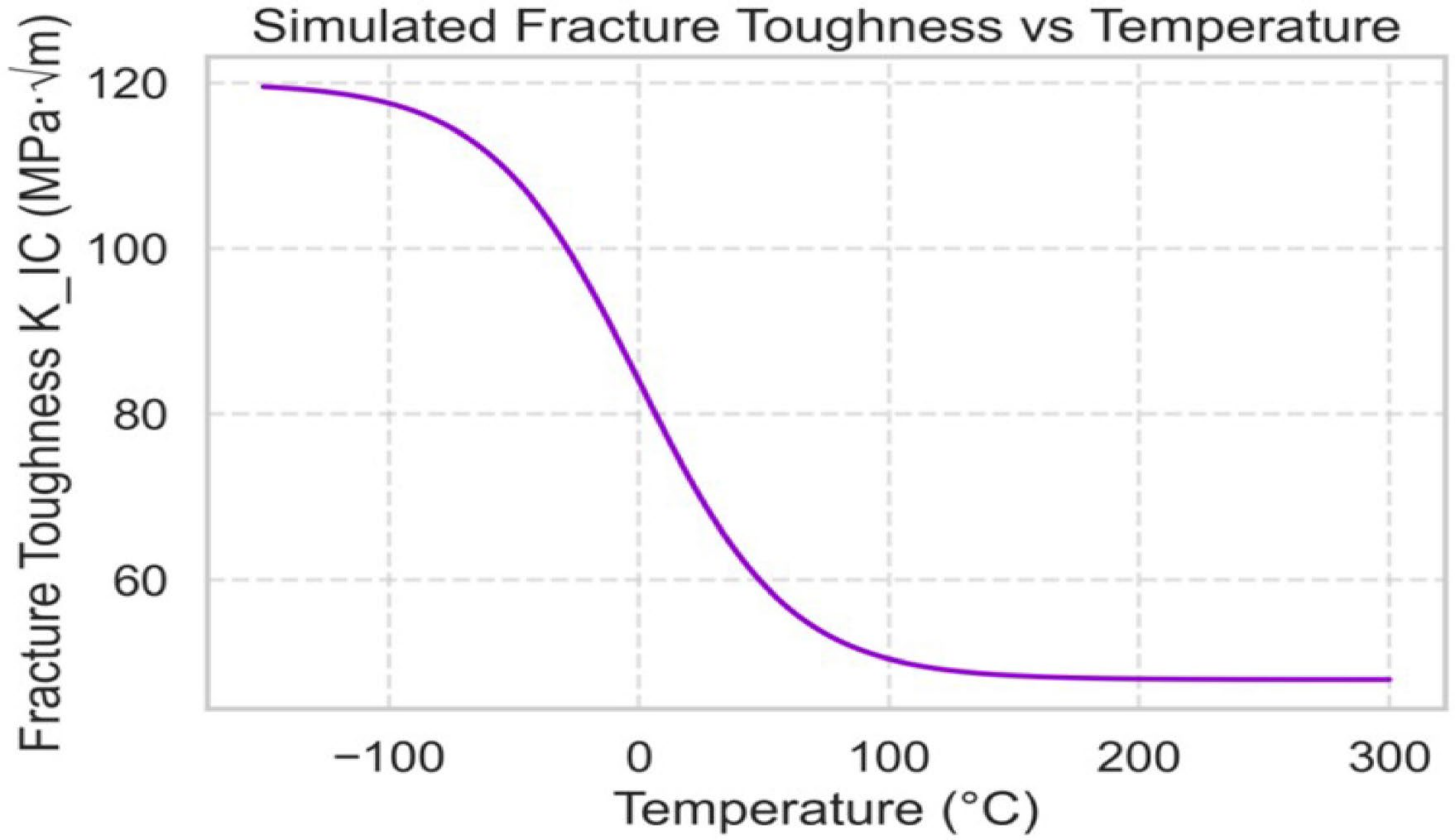
Curve depicting the variation in fracture toughness (K_IC_) across a temperature range.

Paris’ law for fatigue crack propagation was incorporated into the analysis, and Fig. 5; presents an overall view of the simulated crack growth as a function of the number of loading cycles. The logarithmic scale reveals three distinct stress regimes. At a low number of cycles, crack growth remains slow and nearly stable, corresponding to the crack initiation and early propagation stage. As the number of cycles increases, a transition toward accelerated crack extension is observed. Finally, at a high number of cycles, a sharp increase in crack length occurs, indicating unstable crack propagation that leads to rapid failure upon reaching the critical zone^22^. This behavior is consistent with classical fatigue and fracture mechanics theories and highlights the decisive role of cyclic loading in structural integrity^23^. The results emphasize the importance of monitoring crack propagation to accurately predict fatigue life and prevent catastrophic failure in engineering components. The stress-strain behavior of the metals under study, which are mostly ductile with considerable plastic deformation before fracture, is shown in Fig. 6. Because of the perfect balance between strength and ductility, which improves resistance to crack initiation and propagation, the large area under the curve (2420.6) indicates great energy absorption capacity and overall toughness. The ability of the material to release pre-fracture energy through plastic deformation processes like slippage, twinning, and stable crack propagation is also represented by the curve. The stability zones that have been seen show that internal energy dissipation processes are exhausted. This behavior is typical of ductile materials, where energy absorption before critical crack development is greatly influenced by plastic deformation (Fig. 7).

The simulated evolution of tear strength (K_IC_) as a function of temperature is shown in this image. The material shows high K_IC_ values at low temperatures, suggesting strong resistance to crack propagation. A progressive loss of strength is seen as the temperature rises, which results in a decline in tear resistance. This transition, which may be associated with an elastic-brittle transition or a modification of the high-temperature deformation and degradation mechanisms, is indicative of a change in the mechanical behavior of the material. The sensitivity that builds up during fracture initiation and propagation is indicated by the higher-temperature KIC, which is concerning for the long-term service life of components impacted by harsh thermal conditions.

**Fig. 8:**
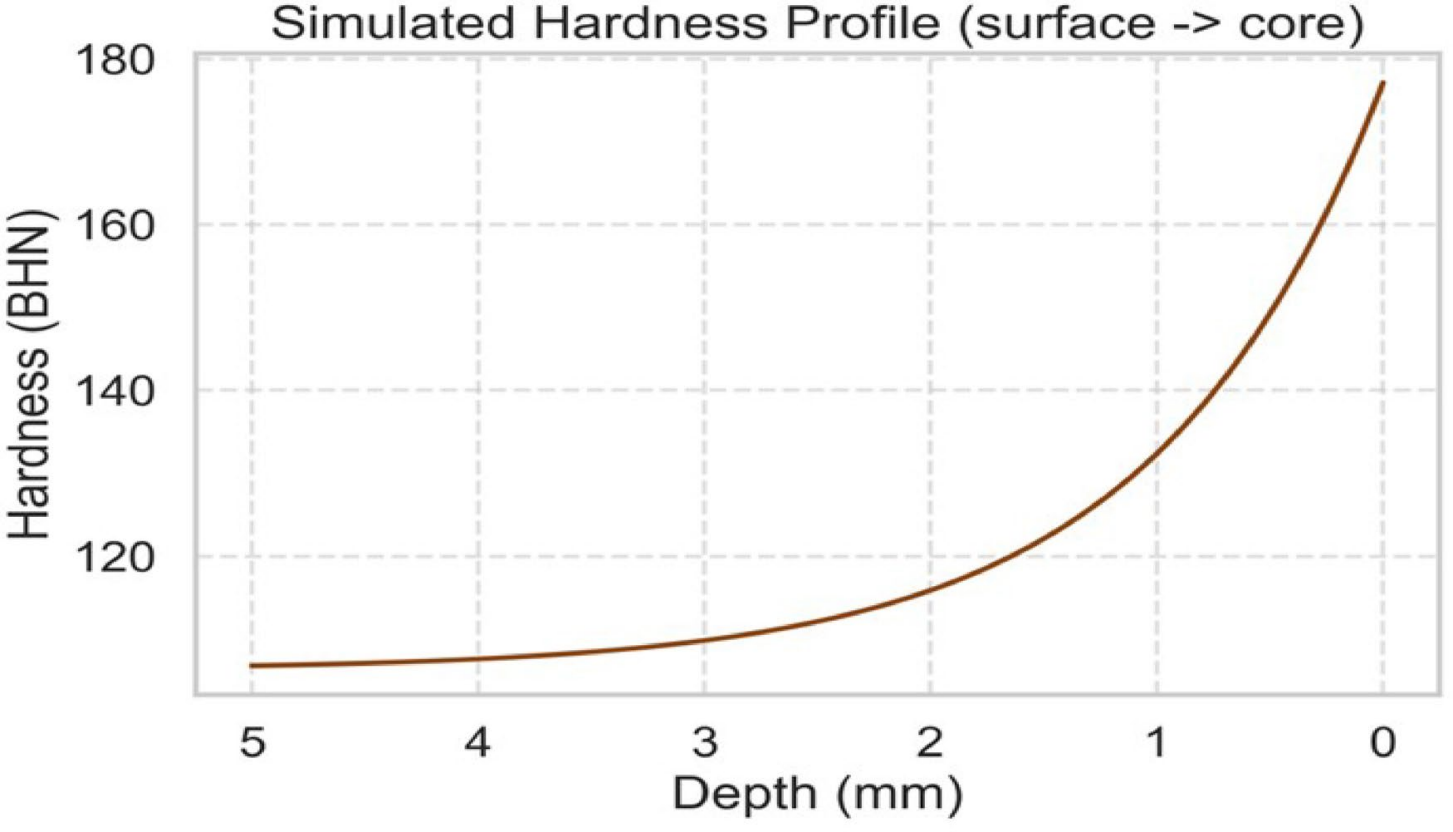
Hardness profile vs. depth.

The evolution of the material hardness (BHN) as a function of depth from the surface to the bulk is shown in Fig. 8. Near the surface, there is a high hardness that progressively diminishes as depth increases.

Three separate zones are shown in the hardness profile (BHN) over a depth of around 5 mm:

- *Zone I* Surface layer (0–1 mm): High hardness (~ 170–180 BHN) that sharply decreases with depth. dominated by substantial residual stresses, high dislocation density, and work hardening. Wear resistance and crack start resistance are improved in this zone.
- *Zone II* Transition zone (1–3 mm) with a more modest decline and intermediate hardness (~ 130–150 BHN). has a combination of mechanisms, including stress relaxation, partial plastic deformation, and residual hardening. It guarantees that the ductile core and the hardened surface are mechanically continuous.
- *Zone III* Core (3–5 mm) with a uniform microstructure and low, steady hardness (~ 105–115 BHN). dominated by energy absorption and ductility, reducing brittle fracture and enhancing general toughness.

**Fig. 9:**
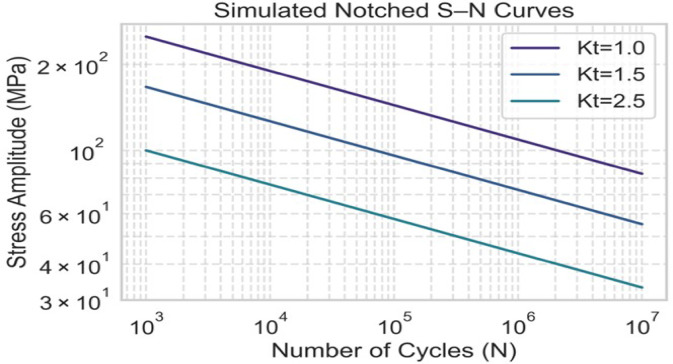
Stress amplitude versus number of cycles for different notch radii or material conditions.

The S–N curves (stress vs. number of cycles) for various values of the stress concentration factor Kt are shown in this picture. As the number of cycles increases, the stress amplitude falls for all configurations, which is consistent with conventional fatigue behavior. Higher Kt levels have been found to significantly shorten fatigue life. As a result, materials or geometries with high stress concentrations are more vulnerable to crack initiation and cyclic damage. These findings highlight how important geometry, local stress conditions, and fatigue damage mechanisms are in determining a component’s service life (Fig. 9).

**Fig. 10:**
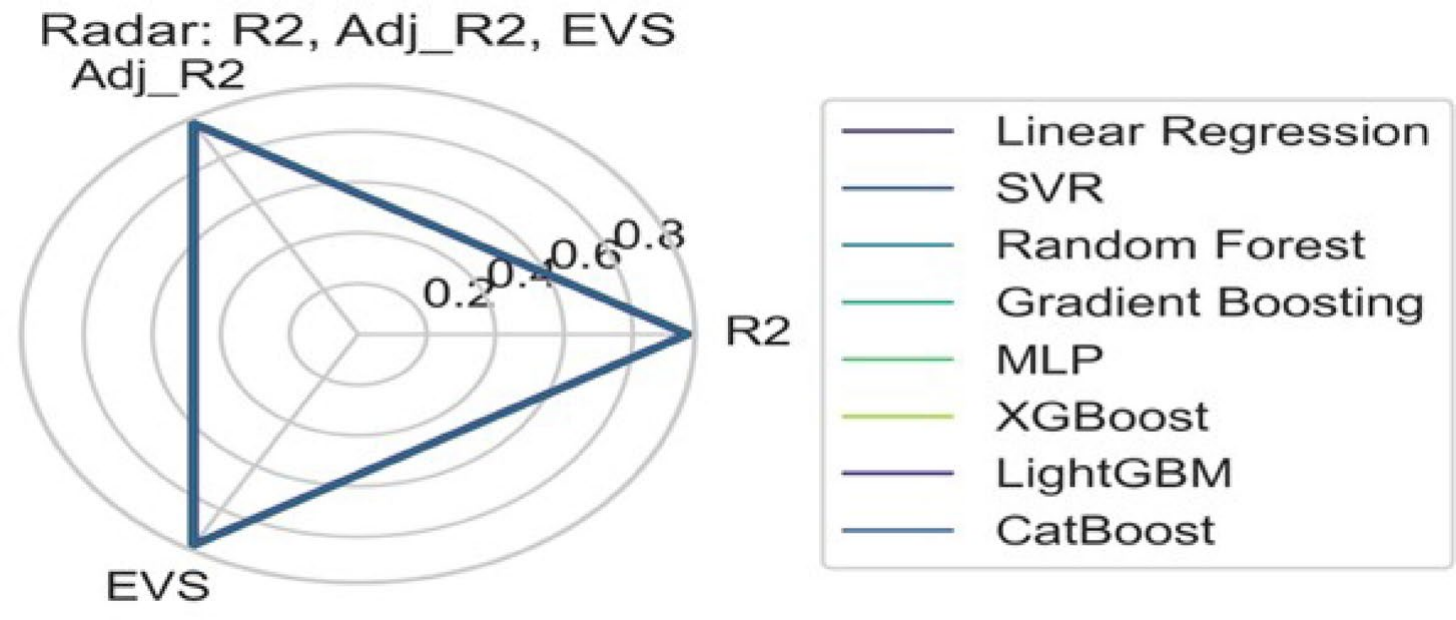
Model performance comparison (radar plot).

The curve highlights the three characteristic regimes of mechanical behavior: the elastic region (reversible deformation), followed by the plastic region (permanent deformation), and finally the strain-hardening stage. The true stress continues to rise even after necking begins because it is calculated using the progressively decreasing actual cross-sectional area. This representation shows the material’s ability to sustain large plastic deformations before failure. The slope of the final segment reflects the intensity of strain hardening, which results from the accumulation and interaction of dislocations that increase the material’s resistance. The radar Fig. 10 chart’s axes each represent a performance indicator (R2, RMSE, MAE, and EVS) that captures a distinct facet of the predictive quality of the model. Stronger overall performance is shown by a model that covers a greater area on the chart. The inherent trade-offs are highlighted in this picture. For example, a model may show a high R² (excellent ability to reproduce global trends) while maintaining a relatively high RMSE, indicating considerable point wise prediction mistakes. As a result, the radar chart shows which model provides the best overall balance between accuracy, stability, and explanatory power in addition to which model performs exceptionally well in a particular parameter.

**Fig. 11:**
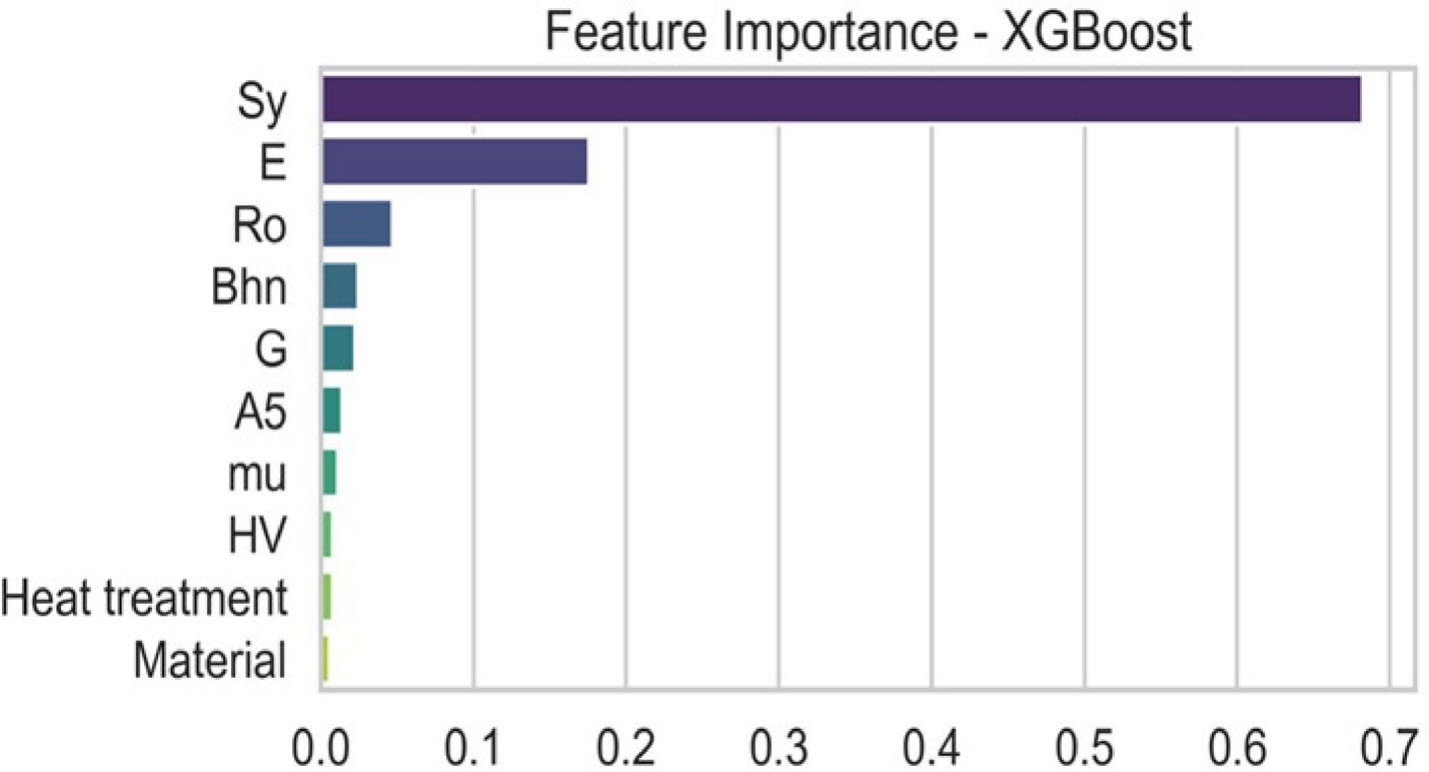
Feature importance—XGBoost Model.

**Fig. 12:**
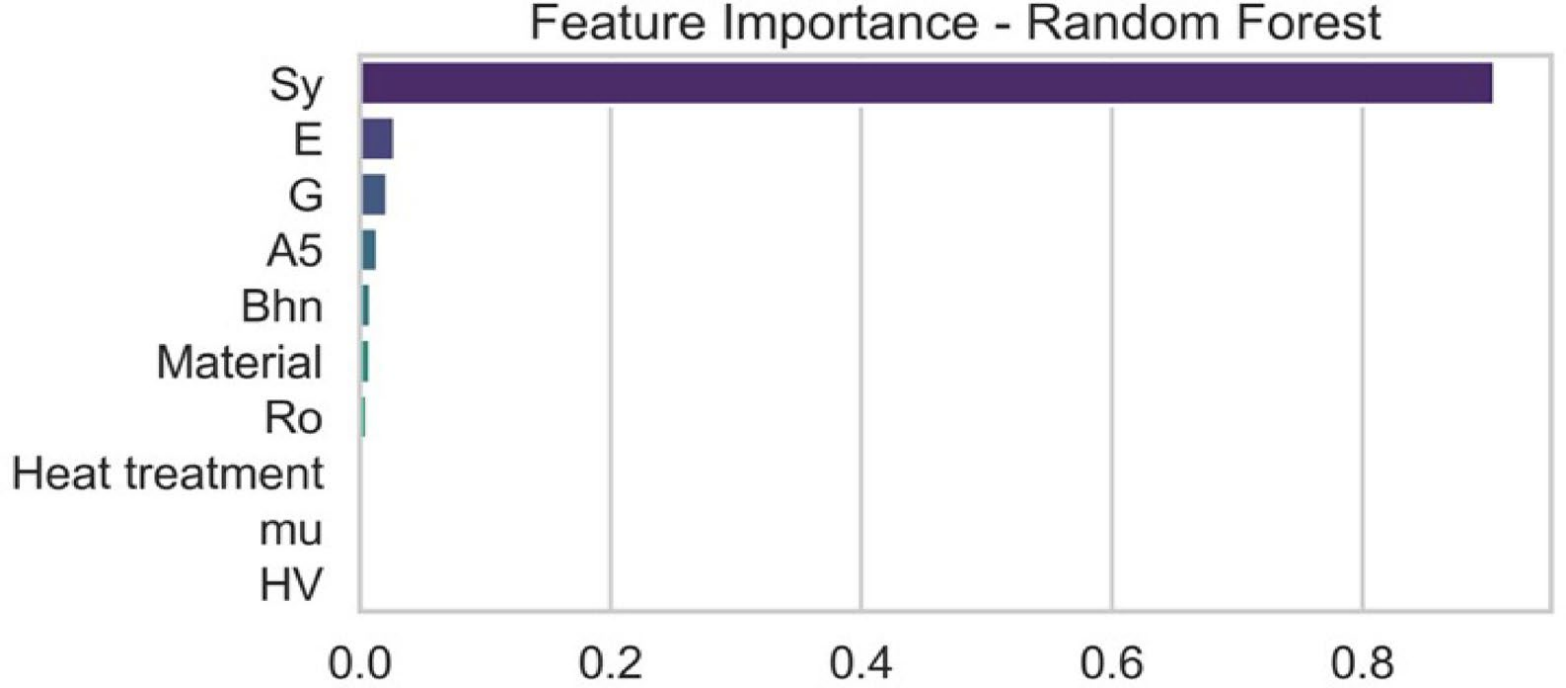
Feature importance—Random Forest model.

**Fig. 13:**
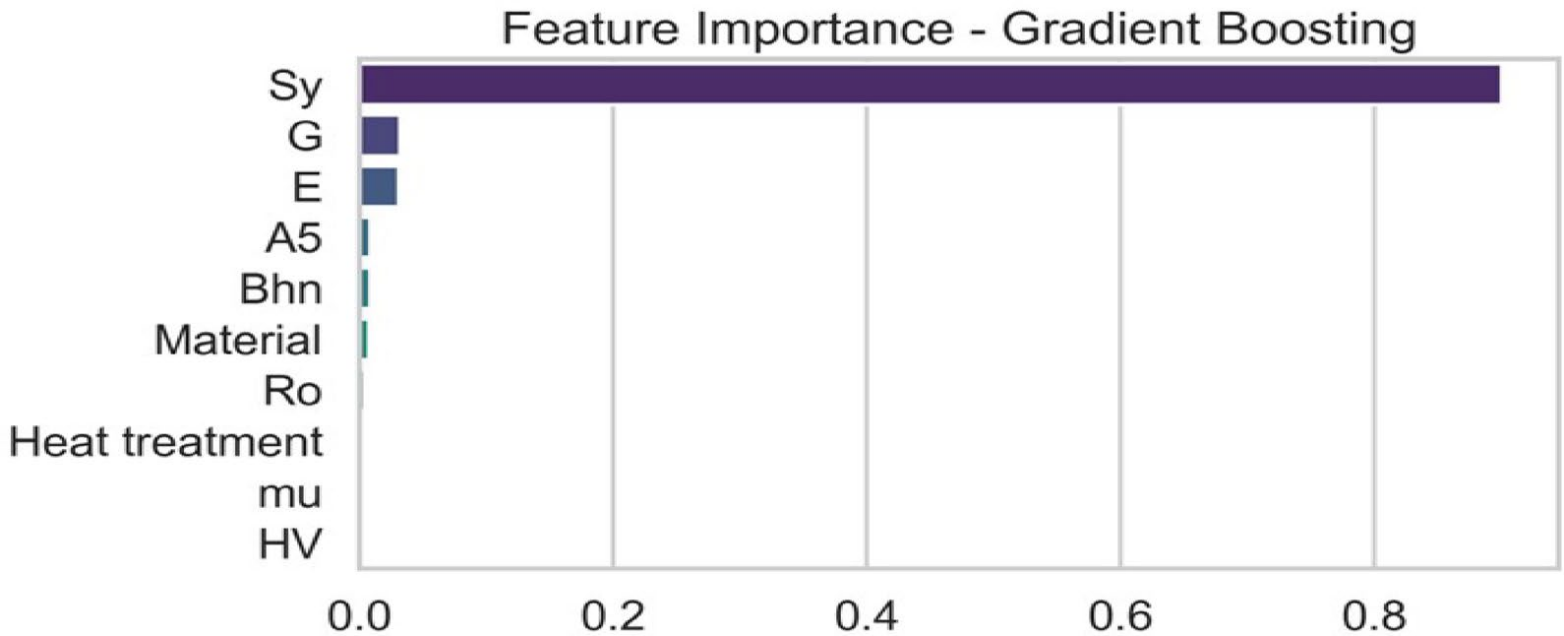
Feature importance—Gradient Boosting model.

Figures 11, 12 and 13 show how various machine-learning models’ predictions are affected by input factors. The most important aspects controlling the simulated mechanical response are the most highly ranked parameters, which are usually fundamental mechanical characteristics like hardness, chemical composition, Young’s modulus, or grain size. Recurring high-impact factors can be found by comparing the outputs of the three algorithms, proving their basic physical involvement in mechanisms of strengthening or harm (e.g., the Hall–Petch effect related with grain-size refinement). Variations in the variable ranks also show how sensitive each model is to nonlinear interactions in the dataset, demonstrating how Random Forest, Gradient Boosting, and XGBoost capture different facets of the data’s underlying structure.

**Fig. 14:**
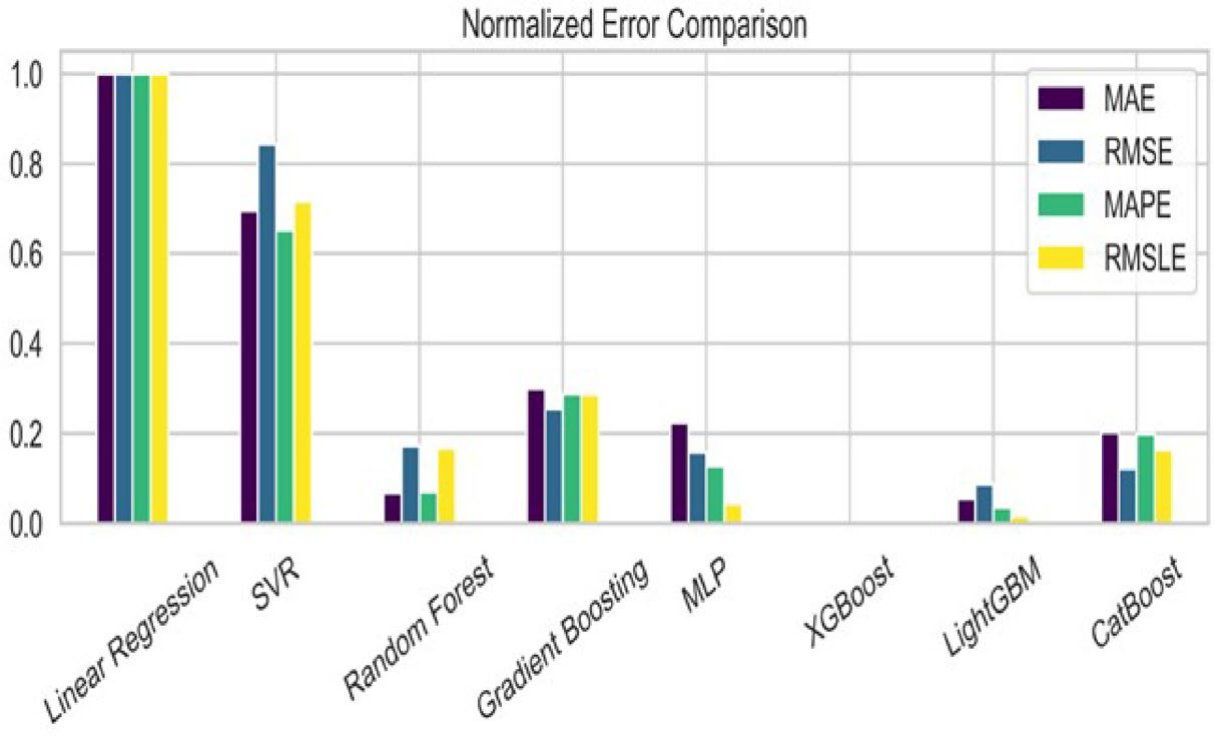
Bar chart comparing error metrics (MAE, MSE, RMSE, etc.) across multiple machine learning algorithms.

**Fig. 15:**
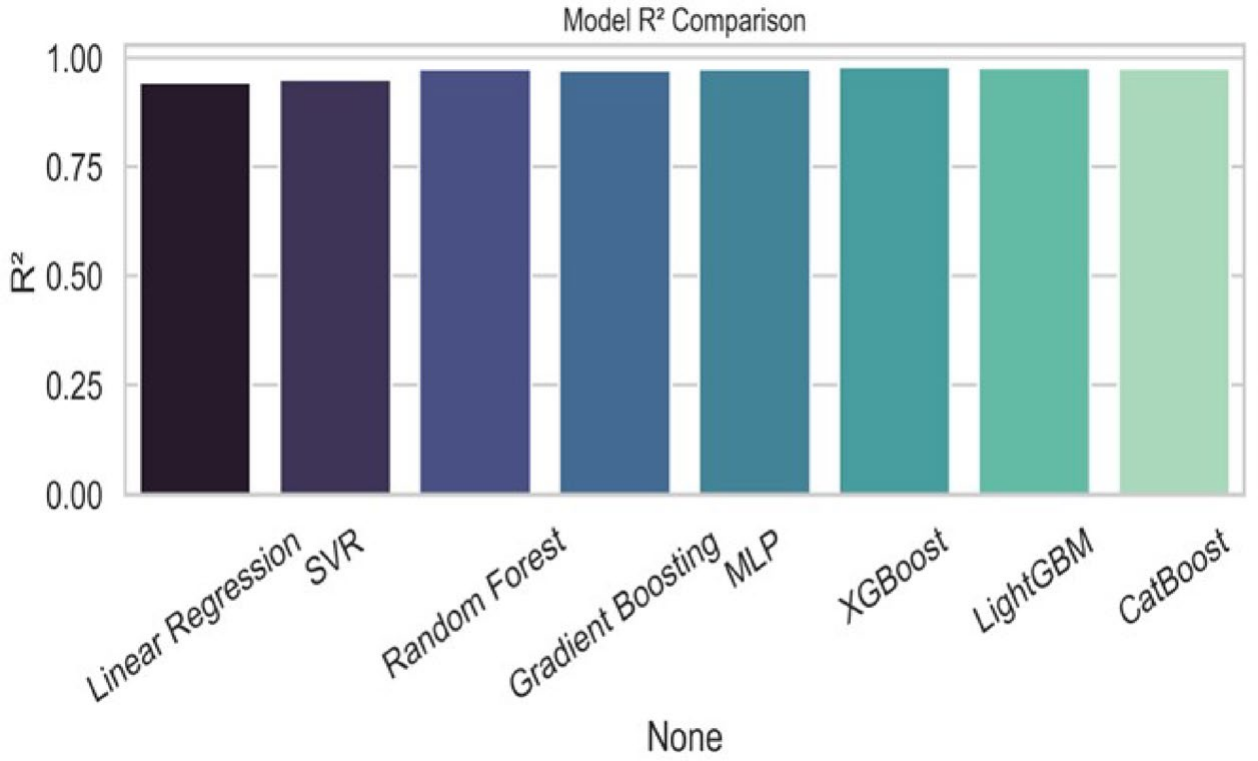
Comparison of coefficient of determination values to evaluate the accuracy of each predictive model.

A comparison of the regression models applied to the dataset under investigation reveals clear and significant differences in prediction efficacy and robustness. Ensemble learning methods, particularly modern boosting algorithms, consistently outperform the other models examined. XGBoost, LightGBM, and CatBoost have the highest coefficients of determination (R2) and the lowest prediction errors (RMSE, MAPE, and RMSLE). proving their excellent ability to capture intricate interactions and non-linear connections between input variables. With the lowest RMSE (44.25), RMSLE (0.0986), and MAPE (0.0654) among various techniques, XGBoost offers the best overall performance, demonstrating strong prediction accuracy and stability over the whole target value range. Multilayer perceptron (MLP) and Random Forest models also perform satisfactorily, demonstrating their efficacy in simulating complex systems. They may not be making the best use of the underlying data structure, though, as their predicted accuracy is still marginally lower than that of boosting-based models. On the other hand, conventional models like support vector regression (SVR) and linear regression have obvious drawbacks. Despite having a good R2 value, linear regression has a significantly larger RMSE (73.80), which indicates that it is unable to accurately capture the dataset’s inherent non-linearity. In a similar vein, SVR’s accuracy is only mediocre and it falls short of ensemble-based methods.These results indicate that the relationships governing the studied system are highly non-linear and involve complex variable interactions that cannot be effectively captured by classical regression approaches. Boosting algorithms outperform other models because they iteratively reduce residual errors and adapt to local data patterns, leading to improved generalization and robustness. The superior performance of XGBoost highlights its ability to balance model complexity and regularization, thereby avoiding overfitting while maintaining high predictive accuracy.Consequently, boosting-based models particularly XGBoost are the most appropriate tools for predictive modeling in this context. Their effectiveness makes them especially suitable for applications involving the prediction of material properties or complex physico-mechanical behaviors, where accuracy, stability, and robustness are critical requirements.The R² coefficient represents the proportion of variance in the target variable that is explained by the model. A value close to 1 indicates strong predictive capability (Table 3). Allows models to be ranked according to their ability to capture the underlying physical trends. Non-linear models such as XGBoost and Random Forest generally outperform linear models, confirming the inherently non-linear nature of the mechanical phenomena studied. In the quantitatively compares prediction errors using MAE, RMSE, and MSE presented in Figs. 14 and 15. The best-performing models are those with the lowest error values. A large difference between RMSE and MAE indicates high sensitivity to extreme values (outliers). This comparison highlights which models handle non-linearity, data dispersion, and experimental noise most effectively.

**Table 3:** Performance comparison of machine learning regression models.

Models	R2	RMSE	MAPE	RMSLE
Linear regression	0.9940	73.8098	0.1307	0.1695
SVR	0.9509	69.1493	0.1080	0.1493
Random Forest	0.9750	49.3540	0.0700	0.1104
Gradient Boosting	0.9724	51.8012	0.0842	0.1189
MLP	0.9754	48.9371	0.0737	0.1018
XGBoost	0.9799	44.2558	0.0654	0.0986
Light GBM	0.9774	46.8446	0.0678	0.0997
CatBoost	0.9765	47.8501	0.0784	0.1102

To further evaluate the robustness and generalization capability of the developed models, performance metrics were assessed on both training and test datasets, as presented in Table 4. The results show that all models maintain consistent performance across both datasets, with only slight differences between training and test metrics. This indicates that the models do not suffer from significant overfitting and are able to generalize effectively to unseen data. In particular, XGBoost demonstrates stable and reliable performance, confirming its suitability for predicting complex nonlinear relationships in metallurgical systems.

**Table 4:** Performance comparison of machine learning models on training and test datasets.

Model	*R*^2^ (train)	*R*^2^ (test)	RMSE (train)	RMSE (test)
Linear regression	0.996	0.9940	65.00	73.81
SVR	0.960	0.9509	60.00	69.15
Random forest	0.982	0.9750	42.00	49.35
Gradient boosting	0.980	0.9724	45.00	51.80
MLP	0.981	0.9754	43.00	48.94
XGBoost	0.989	0.9799	35.00	44.26
LightGBM	0.985	0.9774	38.00	46.84
CatBoost	0.984	0.9765	39.00	47.85

**Fig. 16:**
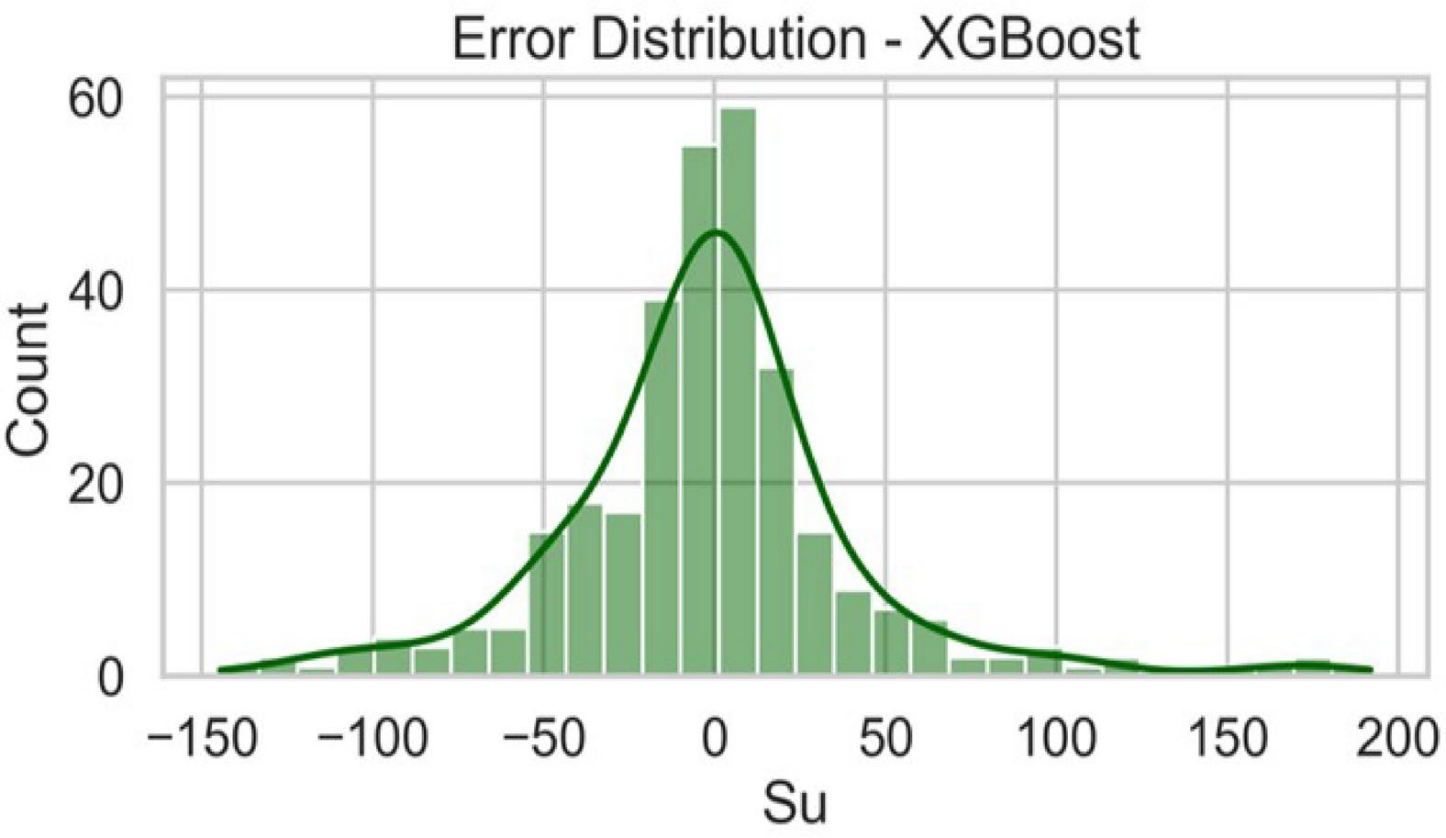
Error distribution – XGBoost Model.

**Fig. 17:**
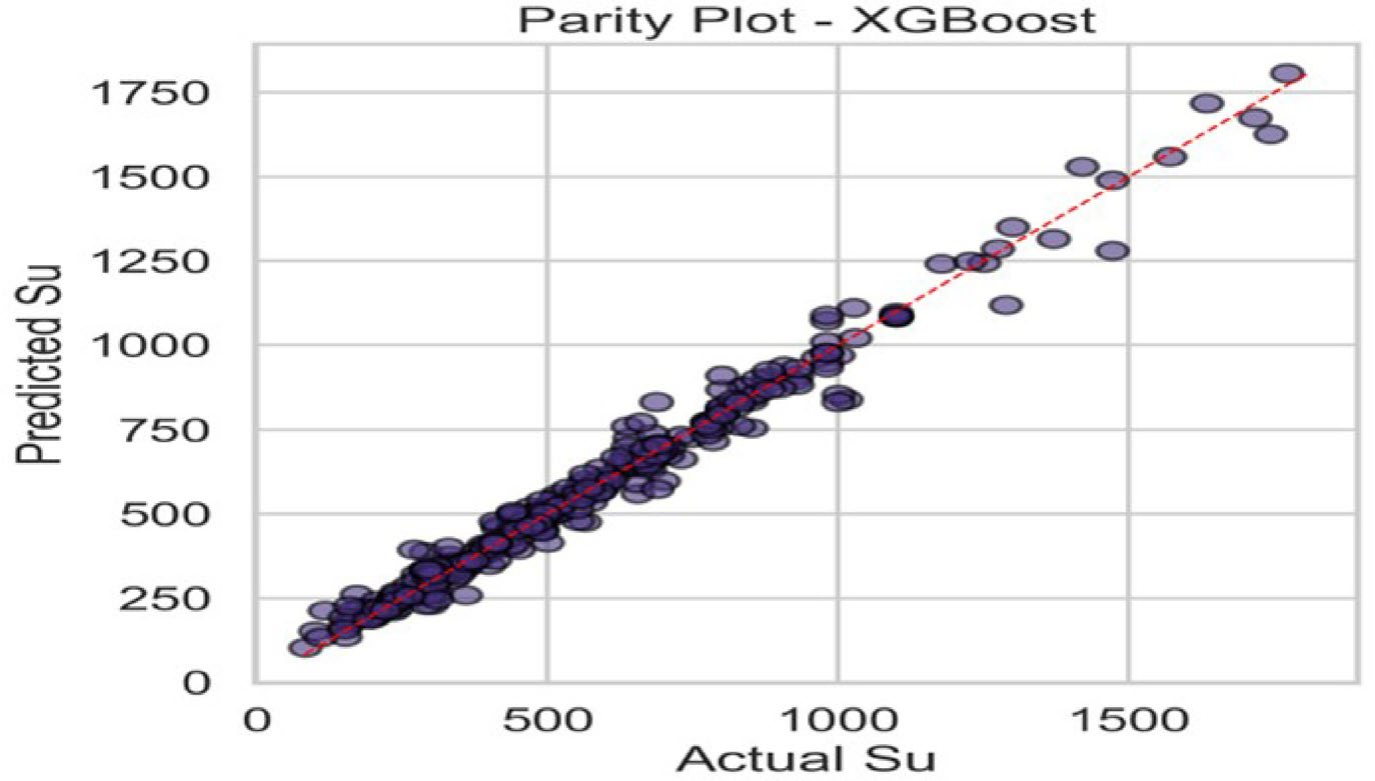
Scatter plot comparing predicted values versus actual values to assess model fitting quality.

The distribution centered around zero indicates that the XGBoost model exhibits no systematic bias, meaning it neither consistently overpredicts nor underpredicts. The narrow spread of the error distribution reflects high overall precision, corresponding to a low standard deviation of residuals. The smooth density curve suggests that the errors follow an approximately normal distribution, which is a strong indicator of the model’s statistical robustness. Moreover, the absence of heavy tails implies that extreme errors are rare, demonstrating the stability and reliability of the algorithm (Fig. 16). The dense alignment of points along the diagonal confirms that the model accurately reproduces the observed values. Deviations from the reference line highlight areas where the model’s precision diminishes. The lack of any systematic pattern (no curvature or bias) indicates that the model generalizes well (Fig. 17). The dense alignment of points along the diagonal demonstrates that the model accurately predicts the observed values. Deviations from the reference line pinpoint regions where the model’s predictive accuracy diminishes. The absence of systematic patterns, such as curvature or bias, indicates that the model generalizes effectively across the data.

The random distribution of residuals around zero indicates that the model exhibits no systematic bias. The absence of any discernible trend reflects good stability. The few outlying points correspond to challenging predictions, often associated with extreme or noisy data.

**Fig. 18:**
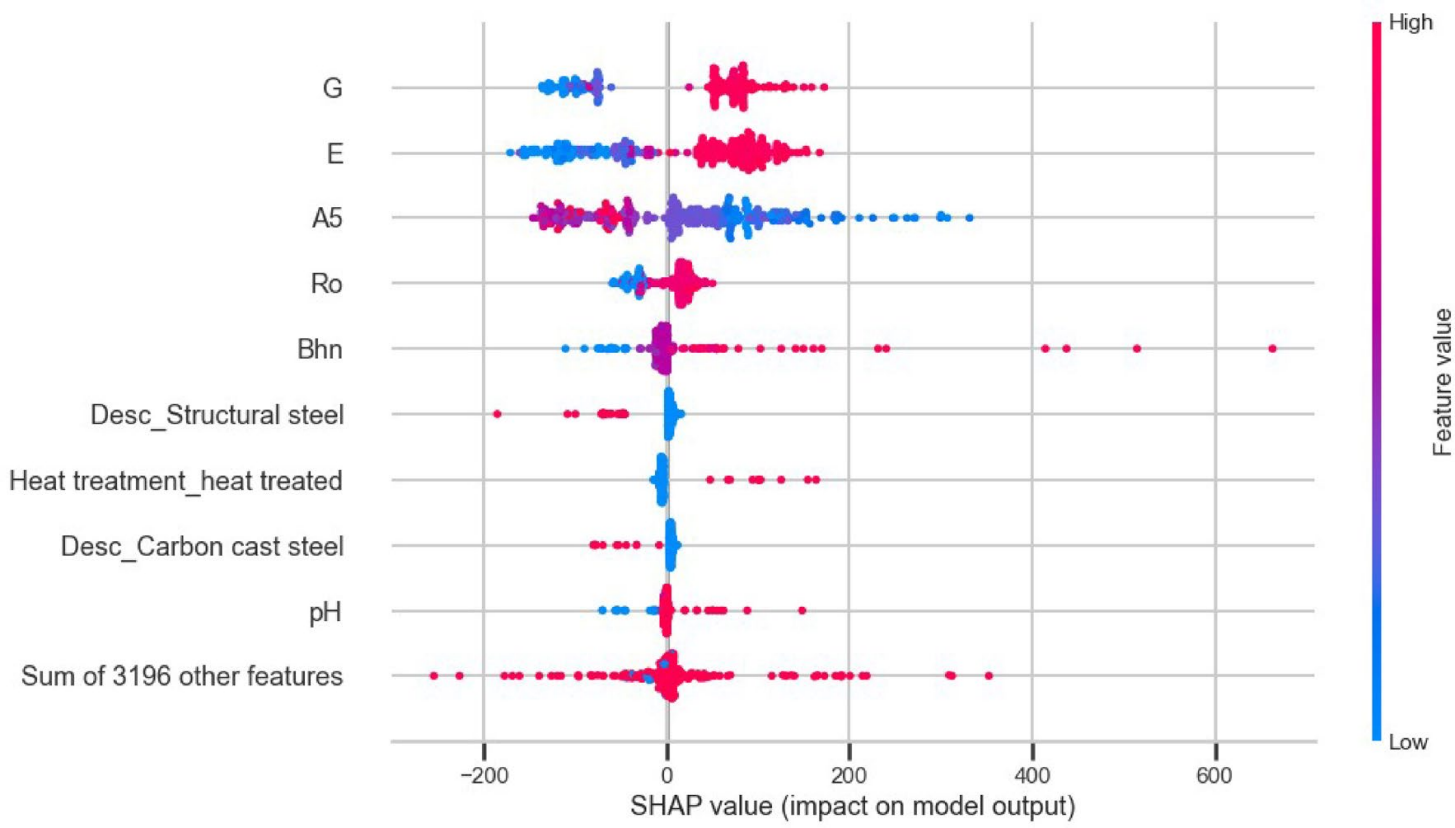
SHAP summary plot (Beeswarm plot).

The model emphasizes how input features affect ultimate tensile strength (Su) prediction in different ways. The most important factors are determined to be mechanical characteristics such the shear modulus (G), elastic modulus (E), elongation at fracture (A5), Brinell hardness (BHN), and Ro. High values of G and E indicate a significant positive contribution to Su, which is in line with traditional mechanical theories that relate tensile strength to material stiffness. The interaction between ductility and resistance to plastic deformation in determining ultimate strength is reflected in the combined effect of A5 and BHN. The dispersion of data points suggests non-linear and heterogeneous effects, implying that the influence of certain features is dependent on how they interact with other structural and mechanical parameters. Steel type and heat treatment-related categorical variables show a lesser but still discernible impact, perhaps due to their indirect impact on microstructural evolution. All things considered, the model effectively represents the linear and non-linear relationships controlling steel’s tensile strength, demonstrating its dependability for predictive and interpretive applications. Figure 18 presents the global SHAP decision plot, illustrating how the prediction progressively evolves from the baseline value to the final output through cumulative feature contributions. The dominant features induce the largest shifts, confirming their governing role in the mechanical response, while intermediate variables introduce interaction-driven adjustments. The final features provide minor refinements, leading to a stable and physically consistent prediction. The limited dispersion of trajectories highlights the robustness and generalization capability of the model. The local explanation of the model prediction for Sample 0 is depicted in the SHAP waterfall plot. The anticipated strength is greatly increased by positive contributions from the shear modulus (G), elastic modulus (E), and elongation at fracture (A5), underscoring the importance of material stiffness and deformation capacity. Conversely, characteristics like heat treatment (heat treated) and Brinell hardness (BHN) have a negative impact, indicating potential trade-offs between hardness, ductility, and overall tensile performance. This local interpretation validates that the XGBoost model provides a physically consistent and comprehensible prediction framework by capturing minor, sample-specific interactions among mechanical and processing parameters in addition to global trends.

## Conclusion

This work demonstrates that integrating machine learning techniques with mechanics-informed analysis provides a robust and reliable framework for predicting the mechanical behavior of heat-treated multi-metal alloys. The developed models achieved high predictive accuracy, with the best-performing model reaching an R² value of approximately 0.97 and low RMSE, confirming the effectiveness of the proposed approach.

- Advanced ensemble models, particularly XGBoost, achieved superior predictive accuracy, highlighting their ability to capture the complex, nonlinear relationships inherent in metallurgical systems. This confirms the suitability of tree-based ensemble methods for handling heterogeneous materials datasets.
- The incorporation of SHAP-based interpretability revealed that fundamental mechanical parameters such as shear modulus, Young’s modulus, elongation at fracture, hardness, and yield strength play a dominant role in governing ultimate tensile strength, ensuring consistency with established physical principles. This provides strong evidence that the model predictions are physically meaningful and not purely data-driven.
- Beyond prediction accuracy, the study offers valuable insights into deformation and failure mechanisms, including fatigue crack propagation, energy absorption, fracture toughness evolution, and hardness gradients from surface to core. These findings emphasize the critical influence of microstructure, heat treatment conditions, and loading regimes on mechanical performance. Such insights enable a deeper understanding of structure–property relationships in multi-metal alloys.
- Overall, the proposed methodology bridges the gap between data-driven modeling and physical understanding, enabling not only accurate prediction but also informed optimization of alloy design and processing routes. This framework can serve as a practical tool for materials design, reducing experimental cost and accelerating industrial applications.

## Data Availability

Data underlying the results presented in this paper are not publicly available at this time but may be obtained from the author (fatmimessaoud@yahoo.fr) upon reasonable request.

## Appendix

### Sample of the dataset

The dataset presented in Appendix was compiled from multiple open-access literature sources, including published experimental studies on heat-treated alloys^8–12,15–18^.

**Table:**

ID	Material	Heat treatment	Su (MPa)	Sy (MPa)	E (GPa)	G (GPa)	A5 (%)	BHN
1	Steel	Quenched	850	620	210	80	12	180
2	Al Alloy	Annealed	320	250	70	26	18	95
3	Ti Alloy	Aged	900	780	110	42	10	220
